# Editorial: New perspectives and insights on heart rate variability in exercise and sports

**DOI:** 10.3389/fspor.2025.1574087

**Published:** 2025-02-26

**Authors:** Jorge L. Storniolo, Luca Correale, Cosme F. Buzzachera, Leonardo A. Peyré-Tartaruga

**Affiliations:** ^1^ Laboratorio Sperimentale di Fisiopatologia Neuromotoria, IRCCS Istituto Auxologico Italiano, Meda, Italy; ^2^ Human Locomotion Laboratory (LOCOLAB), Department of Public Health, Experimental and Forensic Medicine, University of Pavia, Pavia, Italy; ^3^ Human Performance Laboratory, Department of Public Health, Experimental and Forensic Medicine, University of Pavia, Pavia, Italy; ^4^ Departamento de Biofísica, Facultad de Medicina, Universidad de la República, Montevideo, Uruguay

**Keywords:** autonomic nervous system, training load control, recovery, periodization, exercise

**Editorial on the Research Topic**
New perspectives and insights on heart rate variability in exercise and sports

Heart rate variability (HRV) has increasingly gained recognition in recent years as a significant indicator of both mental and physical health, particularly in response to acute or chronic stressors. During the acute phase, the variability in the elapsed time between successive heartbeats (the RR-interval) tends to decrease due to heightened sympathetic nervous system activation during exercise (1, 2). Once exercise stops, this neural activation subsides, leading to increased RR-interval variability associated with the reactivation of the vagus nerve (parasympathetic system). Conversely, chronic HRV assessments performed without physical stimuli provide insights into the overall balance of the autonomic nervous system (ANS). This balance helps determine whether vagal activity is predominant and indicates physiological readiness for subsequent physical challenges or adaptations from long-term training (3). Therefore, careful monitoring and standardization are critical for ensuring that HRV accurately reflects parasympathetic activity—a process influenced by measurement timing, breathing regulation during assessment, and mathematical techniques used for HRV estimation (4).

Based on this rationale, this Research Topic sought studies evaluating HRV responses following exercise to provide insights into optimal recovery from an ANS perspective. These studies focused on various exercise types, intensities, and volumes, examining responses to both single exercise sessions and long-term training effects. The selected articles aimed to clarify, advise, and guide researchers in both theoretical and practical contexts.

In their article, Sammito et al. narratively reviewed key factors influencing HRV responses and their implications for interpretation. The authors categorized these factors into four main domains: (i) physiological factors, (ii) diseases and health impairments, (iii) lifestyle influences, and (iv) external conditions affecting HRV measurement. They noted inconsistencies in replicating HRV results across different studies, even after accounting for factors such as physical fitness levels (5). As emphasized by the authors, careful consideration is required when analyzing HRV in intra- and interpersonal comparisons, highlighting the significant challenges in using this variable as a reliable intensity marker during training periodization in team and individual sports.

In a related study, Deng et al. indirectly assessed ANS function through the heart rate response (HRr) in patients with varying degrees of obesity during a treadmill-based cardiopulmonary exercise test. HRr was calculated as the difference between maximal and resting HR divided by the difference between maximal and resting oxygen consumption. The severe nature of the test required greater sympathetic activation in obese patients compared to the control group, who exhibited higher HRr values. It aligns with findings from Yadav et al. (6), who reported a strong inverse correlation between body mass index (BMI) and HRV, demonstrating that individuals with higher BMI have lower HRV indices. These findings underscore the potential of HRr as a valuable marker for assessing cardiovascular morbidity in the obese population.

Maximal tests have also been utilized to examine HRV variables in real-time control, as proposed by Sempere-Ruiz et al. Their study employed a nonlinear index of HRV, specifically the short-term scaling exponent alpha 1 of detrended fluctuation analysis (DFA a1), which is based on HR time series correlation properties that vary between 1.5 and 0.5 during resting states and exercise, respectively. During exercise, DFA a1 decreases as the work rate increases. The authors introduced an incremental cycling test to assess the DFA a1's reliability and validity in determining intensity thresholds. They found that the DFA a1 method using 0.75 and 0.5 values is reliable and valid for determining HRV thresholds, especially for power output values during this test. These findings highlighted the validity and reliability of HRV thresholds mirroring ventilatory and lactate thresholds. They stimulated practical implications for exercise testing and prescription in clinical and athletic contexts.

Similarly, engaging in acute HRV control during maximal tests but assessed after strenuous exercise, Wang et al. reported different indices to represent HRV reactivity. The authors assumed each index indicated a specific sympathetic or parasympathetic predominance affecting the ANS balance pre- and post-exhaustive exercise. Their results underlined the significant influence of this protocol on bodily responses (HRV), resulting in decreased ANS activity and increased sympathetic nervous system tone. This also confirms the dose-response intensity concerning a more significant shift toward sympathetic dominance, suggesting that systematic HRV monitoring before and after intense workouts could help practitioners adjust training intensity based on each athlete's recovery capacity.

From a different perspective, Bittencourt et al. utilized the root mean square of successive RR-interval differences (RMSSD) to examine whether customizing autonomic recovery periods between resistance training sessions would result in consistent performance improvements. Older women monitored by the recovery RMSSD index (return to training only when their RMSSD values were above their average) showed improvements in muscle mass, strength, and functional performance responses but no significant differences compared to the control group. Ultimately, this resulted in six more training sessions than the fixed control group. Here, untrained individuals and force production may be confounding factors due to the minimal stimulus needed to enhance force, regardless of the average training frequency. Still, the experimental group experienced more stress from the training regimen, requiring more recovery time than the control group, which trained with a lower volume but greater consistency, not reflecting inferior performance.

The exploratory study conducted by Alessandria et al. aimed to investigate the effects of a stretching program following strength exercises on athletic performance, HRV, heart rate recovery (HRR), and blood lactate clearance in healthy adults. The analysis revealed a significant interaction effect between groups concerning squat repetitions; however, no notable differences in HRV, HRR, or blood lactate concentration were observed. The findings suggest that it might help mitigate muscle fatigue since maintaining the same autonomic response linked to increased force production might indicate “responding well” to a chronic stressor, even because the control group began to show a trend of decreasing performance.

In conclusion, this Research Topic highlights HRV as a recovery index, challenging the notion that “more increase is better” without context. While this perspective may apply at a population level, individual acute responses to interventions or stressors must also consider external factors affecting HRV values.
